# Two-dimensional Bi_2_SeO_2_ and Its Native Insulators for Next-Generation Nanoelectronics

**DOI:** 10.1021/acsnano.4c12160

**Published:** 2025-03-05

**Authors:** Pedram Khakbaz, Dominic Waldhoer, Mina Bahrami, Theresia Knobloch, Mahdi Pourfath, Mohammad Rasool Davoudi, Yichi Zhang, Xiaoyin Gao, Hailin Peng, Michael Waltl, Tibor Grasser

**Affiliations:** † Christian Doppler Laboratory for Single Defect Spectroscopy, TU Wien, Vienna 1040, Austria; ‡ Institute for Microelectronics, TU Wien, Vienna 1040, Austria; § Peking University, Beijing 100871, China

**Keywords:** high-κ dielectric, Bi_2_SeO_2_, 2D semiconductor, native oxide, stability

## Abstract

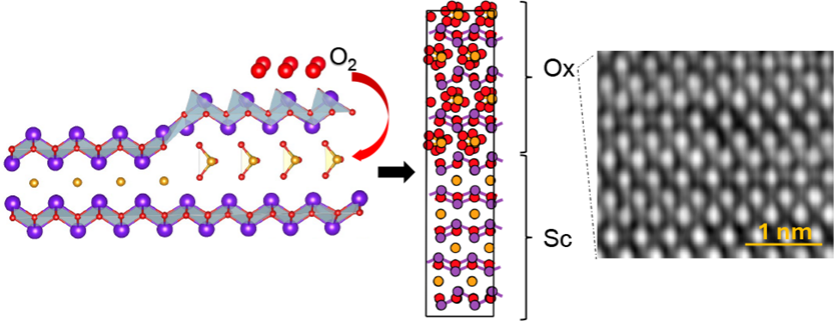

Silicon’s dominance in integrated circuits is largely due to its stable native oxide, SiO_2_, known for its insulating properties and excellent interface to the Si channel. However, silicon-based FETs face significant challenges when further scaled, which inspires the search for better semiconductors. While 2D materials such as MoS_2_, WSe_2_, BP, and InSe are promising, they lack a stable and compatible native oxide. High mobility (812 cm^2^ V^–1^ s^–1^) 2D Bi_2_SeO_2_ stands out in this regard, as it can be oxidized into different forms of Bi_2_SeO_5_, thereby forming compatible high-κ native oxides. Despite growing interest in this material system, a comprehensive understanding of its fundamental properties is lacking. This study uses density functional theory and molecular dynamics simulations to investigate the intrinsic properties of Bi_2_SeO_2_, its native oxides, and its interfaces. Additionally, scanning transmission electron microscopy is employed to complement these theoretical analyses, providing detailed insights into the atomic-scale structure and interfaces of these materials. Building on these findings, we model semiconductor-oxide heterostructures and extract their intrinsic properties. Our results demonstrate that the atomically sharp and clean interface between oxide and semiconductor, the high dielectric constant (>30) of the oxide, and the sufficiently large conduction band offsets between the semiconductor and the most relevant β-phase of its native insulator (1.13 eV for holes and 1.55 eV for electrons) make this material system a strong candidate for future transistor technologies. These properties mitigate the limitations of traditional semiconductors and enhance device performance at the ultimate scaling limit, positioning 2D Bi_2_SeO_2_ as a suitable choice for next-generation nanoelectronics.

## Introduction

Maintaining the continued scaling of silicon technology has become increasingly challenging. This ongoing scaling process has led to soaring costs and highly intricate processing, encompassing over a thousand steps for each wafer. Moreover, as the industry approaches extremely small gate lengths (*L*_*G*_) below 12 nm, scaling encounters an inherent physical barrier, as the channel thickness *t* needs to be decreased accordingly to ensure the device performance remains optimal. A rough guideline is that *t* should be smaller than *L*_*G*_/4 to ensure effective gate control. However, in silicon and other three-dimensional (3D) semiconductors, as the layer thickness is scaled below 4 nm, quantum mechanical effects become increasingly prominent. One critical consequence is a notable reduction in charge carrier mobility due to enhanced interface scattering.^1^ Beyond this, aggressive channel scaling introduces additional challenges, most prominently, a sharp increase in threshold voltage driven by quantum confinement effects. This shift in threshold voltage can severely impact device performance, compromising reliability and power efficiency.^2^

Two-dimensional (2D) semiconductors have attracted a considerable amount of attention due to their exceptional properties. These atomically thin materials can possess significant carrier mobilities, positioning them as ideal candidates for channel materials in scaled FETs.^3,4^ The International Roadmap for Devices and Systems (IRDS) has earmarked 2D materials as a promising option for the next generation of complementary beyond-CMOS devices. The versatility of 2D FETs is also evident from the long list of possible applications. Seamless integration of 2D materials with traditional silicon CMOS chips, especially at the back end of the line, has been demonstrated recently, for example in a graphene-based image sensor array constructed on a CMOS chip.^5^ This heterogeneous integration opens avenues for embedding devices such as 2D material-based single photon emitters,^6^ neuromorphic components like memristors,^7^ and even gas and biosensors.^8−10^ At the front end of the line, the intrinsic properties of these atomically thin semiconductors open options for the integration of high-performance MOS devices.^11,12^ Notably, materials with layered structures can form van der Waals (vdW) interfaces with various compounds.^13,14^ This stacking capability facilitates the engineering of pioneering heterostructures,^15,16^ enabling the creation of novel device architectures.^17,18^ Such innovations can address the Boltzmann limit associated with the subthreshold slope in traditional FETs and pave the way for advanced devices like tunnel^19^ and Dirac source FETs.^20^

Nevertheless, the application of 2D semiconductors faces some challenges,^21,22^ especially regarding gate dielectric integration. Conventional silicon-based FETs typically rely on atomic layer deposition (ALD) of ultrathin amorphous high-κ dielectric HfO_2_ on top of a thin native SiO_2_ layer to improve the interface quality.^23^ Direct deposition of HfO_2_ onto 2D semiconductors presents inherent issues. While using a molecular seeding layer can achieve uniform HfO_2_ dielectrics on 2D semiconductors via ALD, the low dielectric constant of this seeding layer is undesirable. On the other hand, while layered hexagonal boron nitride (hBN) provides a high-quality interface, its process inefficiencies and low-κ make it a less-than-optimal choice for sub-1 nm-EOT dielectrics in integrated electronics.^24−26^ Ionic crystals such as CaF_2_ have attracted significant interest in recent years, but interface quality optimization and fabrication of top-gated FET devices with these materials remain challenging.^24,27^

A notable breakthrough for device-grade dielectrics has recently been achieved through the systematic layer-by-layer oxidation of the high-mobility 2D semiconductor Bi_2_SeO_2_ (812 cm^2^ V^–1^ s^–1^ at room temperature^28^ and up to 47 × 10^4^ cm^2^ V^–1^ s^–1^ at 1.8 K^29^), leading to the formation of an atomically thin gate dielectric, bismuth oxyselenite (Bi_2_SeO_5_).^30^ UV oxidation results in a single-crystalline, atomically flat, nearly lattice-matched interface with the underlying semiconductor.^31^ This smooth interface, characterized by a remarkably low interfacial trap density, significantly improves device performance.^28^ The high dielectric constant of Bi_2_SeO_5_ ensures excellent insulating properties and optimal band alignment with Bi_2_SeO_2_, resulting in devices that exhibit high field-effect mobility (812 cm^2^ V^–1^ s^–1^) and high on/off current ratios (>1 × 10^6^).^28^ Additionally, selective etching of Bi_2_SeO_5_ without compromising the underlying Bi_2_SeO_2_, utilizing advanced UV photolithography,^30^ enables the wafer-scale production of uniform native oxide dielectrics with high-quality interfaces.^31^ This advancement facilitates the fabrication of high-quality planar and FinFET devices with remarkable scalability and performance, such as top-gated 2D FETs with sub-0.5 nm equivalent oxide thickness (EOT) dielectrics that exhibit low leakage current and maintain high insulative quality, meeting the requirements of next-generation nanoelectronics.^32^

Despite these achievements, a comprehensive understanding of the fundamental properties of this material system is lacking, and details such as their precise crystal structures and intrinsic material properties remain unclear. This study investigates the structural, electronic, and dielectric properties of Bi_2_SeO_2_ and its native insulators employing density functional theory (DFT) and molecular dynamics (MD) simulations. These simulations are built on information from detailed experimental characterization using Scanning Transmission Electron Microscopy (STEM). Furthermore, we investigated the impact of strain on both the semiconductor and the oxide to determine the possible range of parameters for a real interface.

## Results and Discussion

The following section first presents an analysis of the structural properties, including the crystal structure and its stability, laying the foundation for understanding the Bi_2_SeO_2_ material system. Next, the electronic and dielectric properties are studied, including the band structure, effective masses, and dielectric constants, providing insight into the material’s intrinsic properties. Finally, semiconductor-oxide heterostructures are analyzed, emphasizing interface quality and band alignment, to underscore their potential for high-performance electronic devices in next-generation nanoelectronics.

### Structural Properties

**Figure 1:**
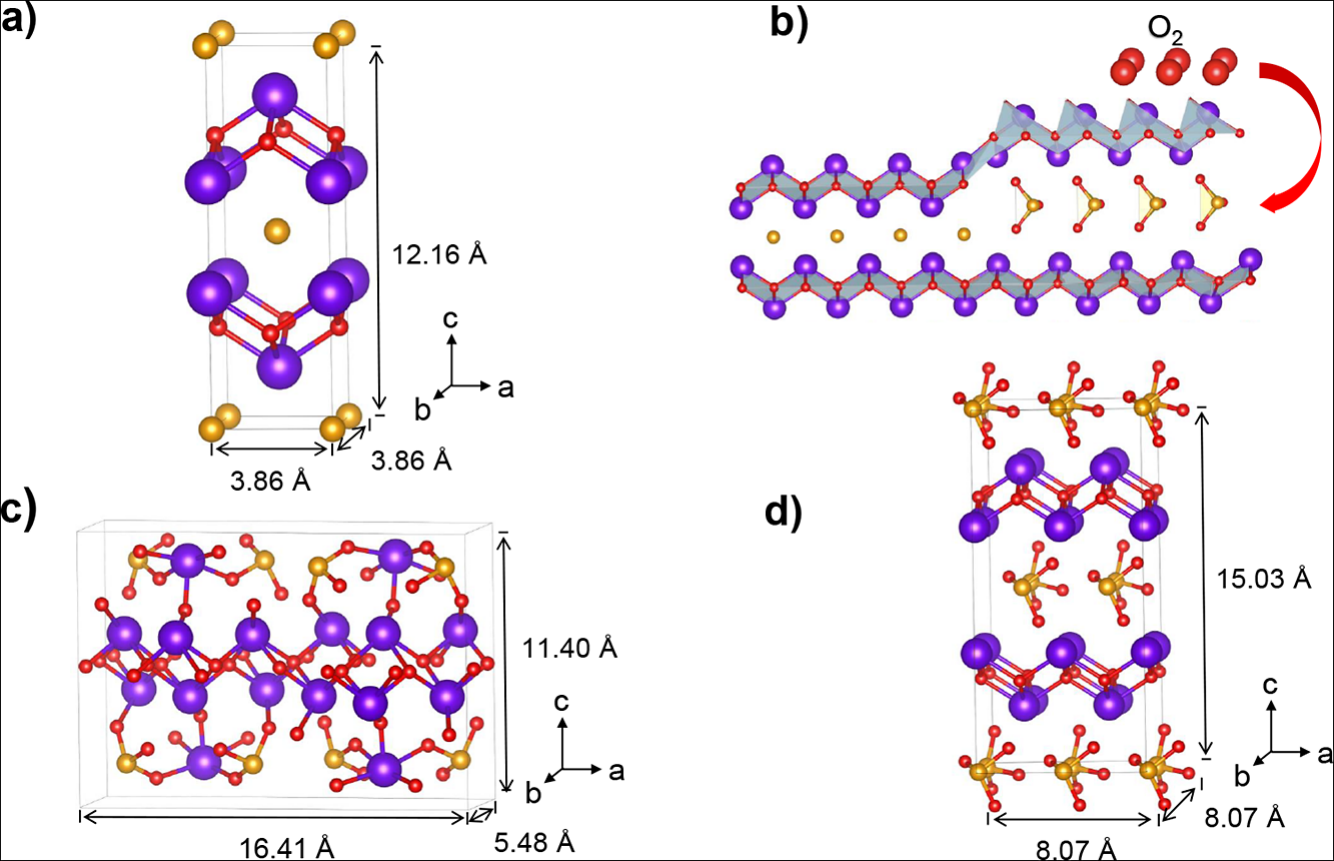
Crystal structures of Bi_2_SeO_2_ and its native oxides. (a) Crystal structure of the Bi_2_SeO_2_ semiconductor, with buckled [Bi_2_O_2_]^+^ layers centrally positioned between planar [Se]^−^ layers. (b) Schematic representation of the oxidation process of the semiconductor, showing the interlayer enlargement between the Bi_2_O_2_ layers after oxidation. (c) Crystal structure of α-Bi_2_SeO_5_ oxide and (d) crystal structure of β-Bi_2_SeO_5_ oxide, highlighting the detailed atomic configuration and lattice dimensions.

The layered oxychalcogenide semiconductor Bi_2_SeO_2_ exhibits the highly symmetric *I*4/*mmm* space group with lattice parameters *a* = *b* = 3.86 Å, *c* = 12.16 Å, and *Z* = 2 (Figure 1a). Similar to many other bismuth-based oxychalcogenide and oxyhalogen materials, Bi_2_SeO_2_ consists of positively charged Bi_2_O_2_ layers and negatively charged Se layers oriented along the *c*-axis.

**Figure 2:**
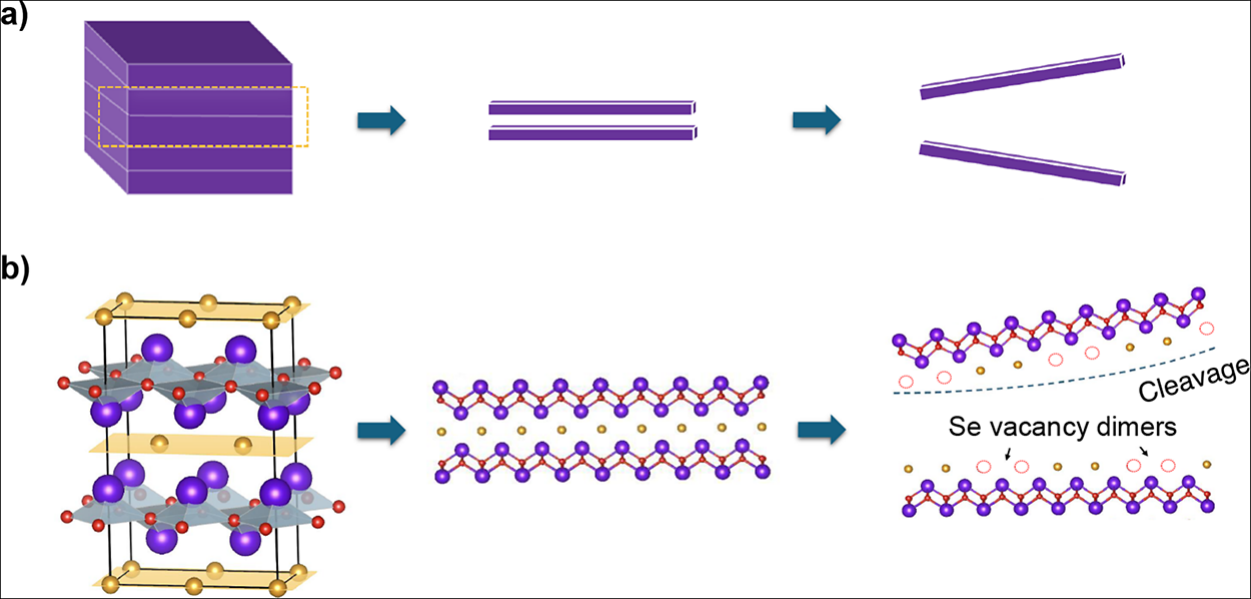
Comparison of vdW and zipper 2D materials. (a) Structure of a typical vdW material, illustrating the presence of a vdW gap. (b) Structure of a zipper material, characterized by the absence of such a gap in its crystal structure and half-occupied surfaces.

Experimental observations and our computational results indicate that Bi_2_SeO_2_ consists of 2D layers but differs significantly from typical vdW 2D materials (Figure 2). This material is categorized as a “zipper 2D” material due to its distinct features, including the absence of a vdW gap in its crystal structure and larger binding energies between layers compared to most vdW materials. Additionally, the half-occupied surfaces, featuring vacancy dimers (as shown in Figure 2b), do not introduce any surface states within the electronic band gap. This contrasts with the typical phenomenon in vdW materials, where surface vacancies would otherwise introduce such states. Notably, the bandgap of Bi_2_SeO_2_ exhibits a thickness dependence typical of layered 2D materials, with a bandgap widening in thinner configurations, largely due to quantum confinement effects, particularly as the material approaches its monolayer form. The monolayer structure consists of centrally buckled [Bi_2_O_2_]^+^ layers, capped by planar [Se]^−^ layers on both surfaces, preserving bulk inversion symmetry.^33,34^

Few-layer Bi_2_SeO_2_ possesses two distinct native oxides,^30,31^ α- and β-Bi_2_SeO_5_, distinguished primarily by their synthesis techniques. The α-Bi_2_SeO_5_ is synthesized through thermal oxidation powder sintering of Bi_2_SeO_2_.^30^ Experimental studies suggest that this phase is thermodynamically stable and forms a dense, well-connected network structure. In contrast, the single-crystalline native oxide termed the β-Bi_2_SeO_5_, is produced via ultraviolet (UV)-assisted intercalative oxidation of Bi_2_SeO_2_ (Figure 1b). In this process, the oxidant sequentially unzips the [Bi_2_O_2_]^+^ frameworks and oxidizes the interlayer Se anions of Bi_2_SeO_2_.^31^ This native β-oxide is stable at low temperatures and undergoes a phase transition at around 380 °C to the thermostable α-phase.^31^

The crystal structure of α-Bi_2_SeO_5_ is well-established^30^ and shown in Figure 1c. After thermal oxidation at elevated temperatures, more oxygen atoms intercalate into the structure, connecting all Se and Bi atoms into a network to form a dense α-Bi_2_SeO_5_ phase with the *Abm*2 space group (*a* = 16.41 Å, *b* = 5.48 Å, *c* = 11.40 Å, *Z* = 8). Unlike conventional metal oxides, which often suffer from oxygen vacancies and variable valence states of metals, α-Bi_2_SeO_5_ is a thermodynamically stable oxide, where the valence states of bismuth and selenium are consistently +3 and +4, respectively.^30^ On the other hand, the crystal structure of β-Bi_2_SeO_5_ is similar to its parent 2D semiconductor (as shown in Figure 1d) with lattice parameters *a* = *b* = 8.07 Å and *c* = 15.03 Å. During the UV-assisted intercalative oxidation process, the [Bi_2_O_2_]^+^ frameworks preserve their structural integrity while undergoing interlayer expansion. Concurrently, the [Se]^−^ anions experience a slight in-plane shift and bond with oxygen atoms, forming [SeO_3_]^−^ tetrahedra sandwiched by the [Bi_2_O_2_]^+^ layers. As a consequence, the crystal structure of β-Bi_2_SeO_5_ largely preserves the original framework, maintaining the overall crystalline integrity and alignment with the underlying 2D semiconductor.^31,32^

#### β-Bi_2_SeO_5_ Structure Determination

Accurate prediction of the oxide crystal structures is pivotal, as it determines their intrinsic properties. However, fully determining the crystal structure of β-Bi_2_SeO_5_ remains challenging given the scarcity of experimental data. While STEM provides high-resolution imaging that precisely resolves the positions of Bi and Se atoms, the significantly lower atomic number of O reduces contrast, introducing ambiguity in accurately determining their positions. Additionally, previously reported XRD data^31^ shows diffraction peaks only for out-of-plane (00*z*) orientations. In the following, we present our systematic approach to determining the crystal structure of β-Bi_2_SeO_5_, combining experimental data and first-principles calculations.

**Figure 3:**
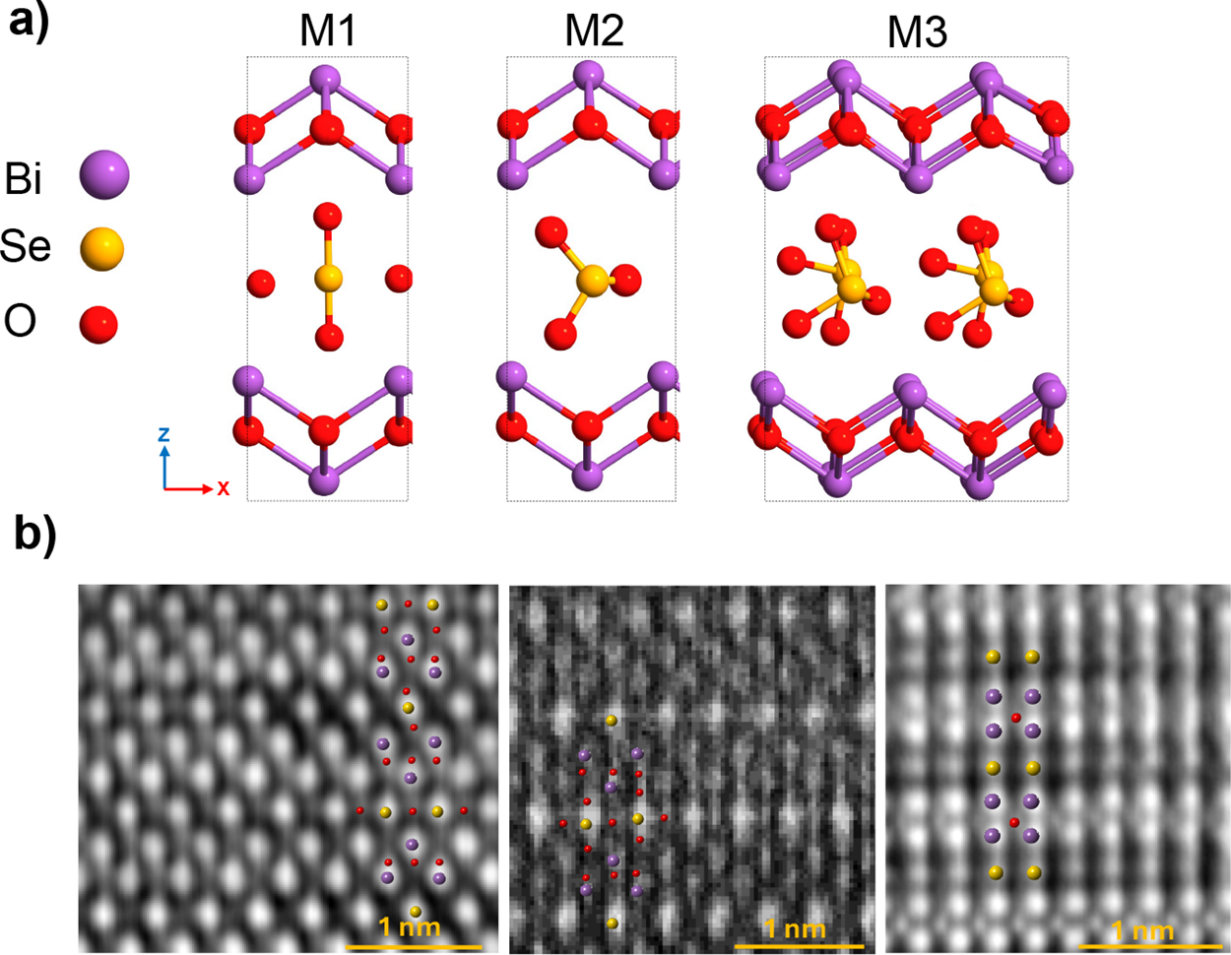
(a) M1: first proposed crystal structure of β-Bi_2_SeO_5_ based on experimental data,^31^ M2: this model results from refining M1 using DFT calculations, M3: this crystal structure is obtained by further refining M2 to enhance its dynamic and thermodynamic stability, achieving the most accurate representation of the crystal structure (shown in more detail in Figure 1d). (b) All three models are consistent with the experimental STEM results. However, distinguishing and determining the position and configuration of the SeO_3_ tetrahedra in the interlayer is challenging. This issue arises because the oxygen atoms in SeO_3_ are essentially invisible in STEM, making it difficult to fully establish the interlayer structure using STEM alone.

Initially, we derived a model based on available experimental data^31^ (M1 in Figure 3a) for the atomic positions and in-plane lattice constants of β-Bi_2_SeO_5_. Using DFT calculations, given the lack of precise data for the interlayer atomic positions, we refined this model iteratively by calculating properties such as bandgap, effective masses, and dielectric constants, and comparing these with experimental values. This iterative process continued until the calculated structural, electronic, and optical properties closely matched the reported experimental values. The refined crystal structure of β-Bi_2_SeO_5_ (M2 in Figure 3a) was commensurate with the primitive cell of the Bi_2_SeO_2_ semiconductor and consistent with the experimental STEM results (Figure 3b). The calculated electronic band gap of this crystal structure (M2) is 3.13 eV (indirect) and is also in good agreement with the experimental value of 3.25 eV (indirect).

**Figure 4:**
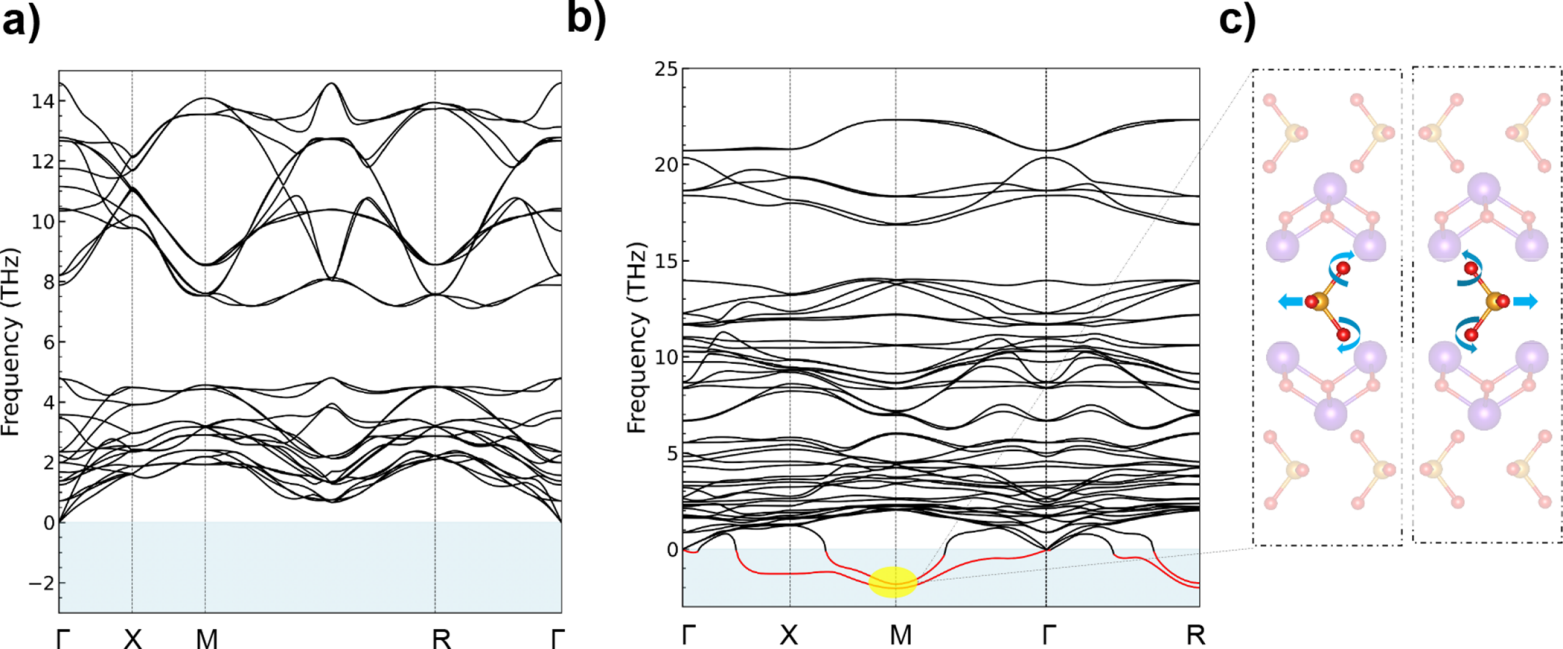
(a) Phonon dispersion of Bi_2_SeO_2_, showing its stable crystal structure with no negative frequencies. (b) Phonon dispersion of the M2 structure of β-Bi_2_SeO_5_, revealing instabilities due to the addition of oxygen atoms and the formation of SeO_3_ tetrahedra between Bi–O layers. (c) Analysis of phonon modes associated with negative frequencies at the M point in the phonon dispersion of β-Bi_2_SeO_5_. negative frequencies of acoustic phonon branches at the M point correspond to the rotational vibration of SeO_3_ atoms, indicating some instability.

**Figure 5:**
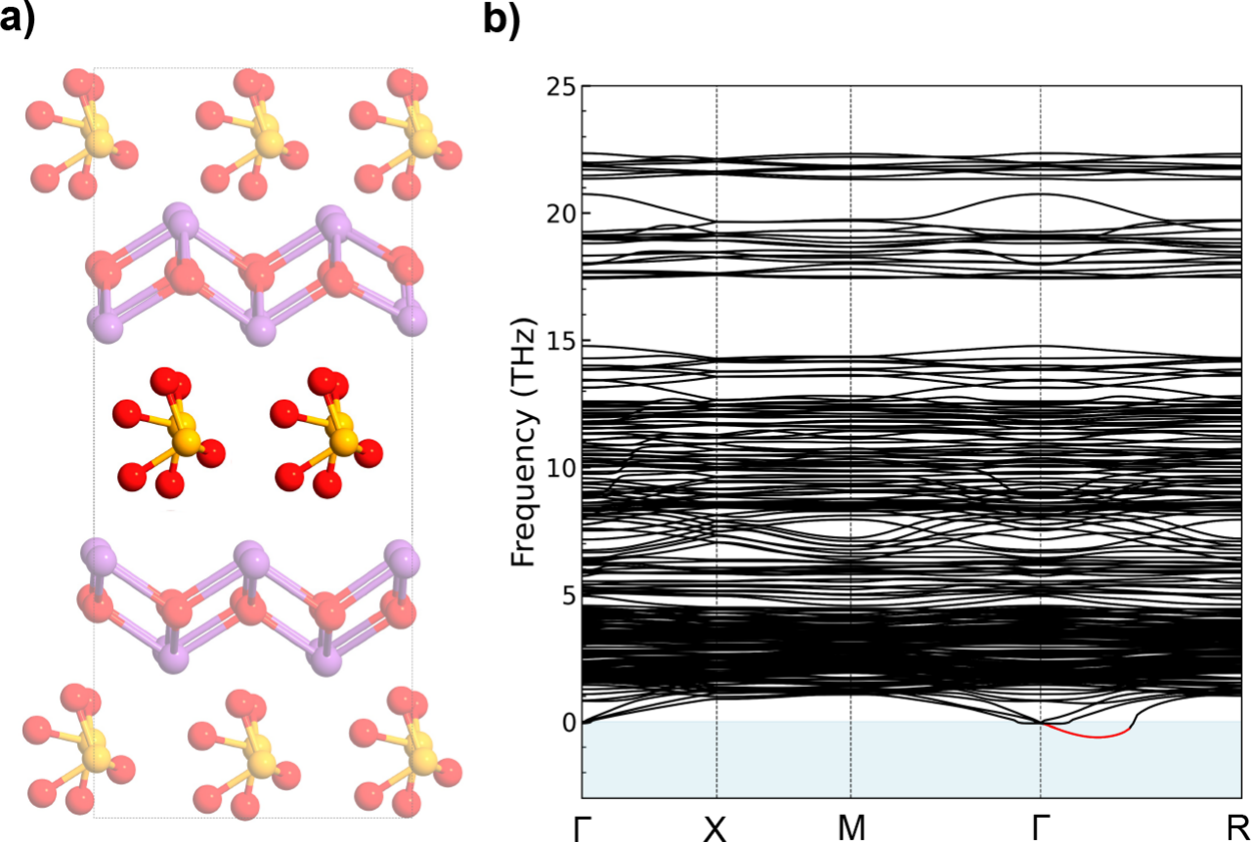
(a) After doubling both in-plane lattice vectors and fully optimizing the structure (M3), the neighboring SeO_3_ tetrahedra indeed are slightly rotated toward each other. (b) Phonon dispersion of the doubled β-Bi_2_SeO_5_ structure, showing a significant reduction in negative frequencies, indicating enhanced stability. However, one acoustic branch still shows negative frequencies between Γ-R, indicating that further rotations in the SeO_3_ tetrahedra in an even larger cell could further lower the total energy.

However, phonon calculations (Phonopy^35^) revealed that the proposed crystal structure of β-Bi_2_SeO_5_ (M2) exhibits partial instabilities due to rotations of the embedded SeO_3_ tetrahedra. As shown in Figure 4, the formation of SeO_3_ tetrahedra between the Bi_2_O_2_ layers during oxidation introduces negative frequencies along the Γ–M and Γ–X paths, suggesting local structural distortions.^36^ Nevertheless, the system retains translational invariance at the Γ point, implying that these imaginary modes are confined to specific vibrational patterns rather than full-scale instability. Phonon mode analysis at the M wavevector (Figure 4b) identifies the origin of instability in β-Bi_2_SeO_5_, attributed to out-of-phase rotational displacements of oxygen atoms within the SeO_3_ units. Eigenvector extraction from the dynamic matrix (Figure 4c) reveals significant atomic displacements, with oxygen atoms surrounding the selenium showing the most pronounced movements. These modes exhibit complex vibrational behavior, characterized by substantial phase shifts in atomic movements. Specifically, the oxygen atoms undergo significant out-of-phase displacements, indicating considerable rotational freedom around the selenium atoms. The extracted eigenvectors confirm this rotational behavior with significant imaginary components in multiple directions, while the selenium atoms display smaller imaginary components, suggesting their relative stability despite the rotational influence of neighboring oxygen atoms. Since the M wavevector lies at the Brillouin zone boundary, the displacement pattern responsible for lowering the energy features opposite signs in adjacent unit cells. Doubling both in-plane lattice vectors and fully optimizing the structure (M3, Figure 5a) result in a slight energy reduction of 18.73 meV per atom, effectively mitigating the impact of negative frequencies (Figure 5b) and improving the overall structural stability.

Despite the presence of partially imaginary modes in the lowest acoustic branch between the Γ and R wavevectors (Figure 5b), indicating that localized dynamic instability is associated with the movement of oxygen atoms within the interlayer and the overall structure remains dynamically stable. The partially imaginary nature of this acoustic branch suggests that these atomic displacements are confined to low-energy vibrational modes and do not imply broader structural instability.

#### Thermodynamic Stability

**Figure 6:**
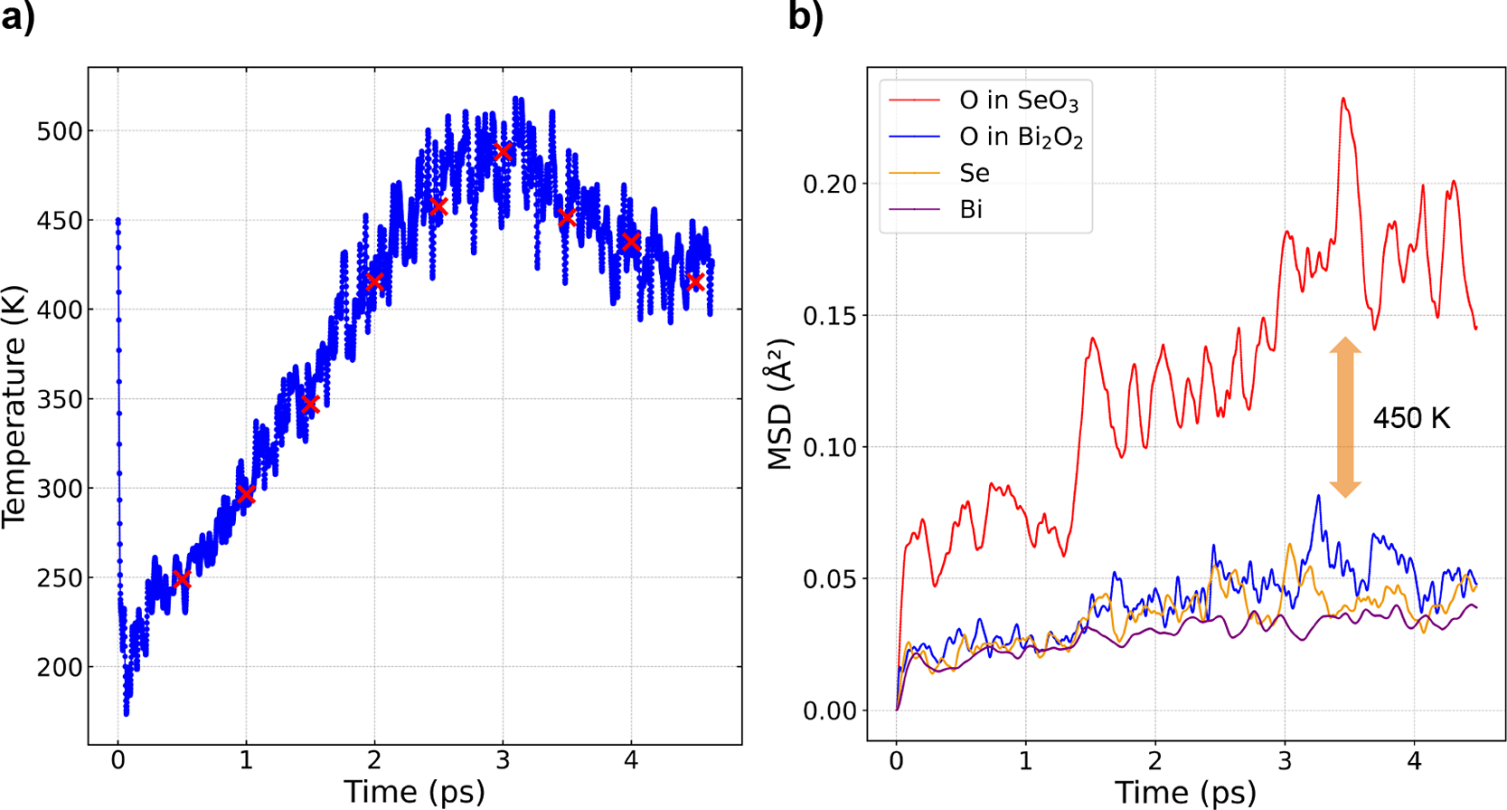
(a) Temperature profile during MD simulations up to 450 K, with red crosses indicating snapshots taken for geometry optimization. All extracted configurations converged to the original structure with only slight differences. (b) MSD of Bi, Se, and O atoms in the SeO_3_ and Bi_2_O_2_ frameworks during MD calculations. The MSD shows that the O atoms in the SeO_3_ tetrahedra displaced considerably more than the other atoms.

To further validate the overall stability, we performed MD calculations up to 450 K (Figure 6). Snapshots of the structure, indicated by red crosses in Figure 6a, were examined via geometry optimization during the MD simulations. All configurations converged to the original structure, confirming that the residual imaginary modes do not evolve into a global phase transition. Additionally, the mean squared displacement (MSD) of the Bi, Se, and O atoms in the SeO_3_ and Bi_2_O_2_ frameworks during the MD calculation reveals that the oxygen atoms in the SeO_3_ tetrahedra exhibit significantly larger displacements compared to the other atoms in the system (Figure 6b). This observation further supports the role of these atoms in the localized dynamic instability, aligning with the phonon analysis results.

**Figure 7:**
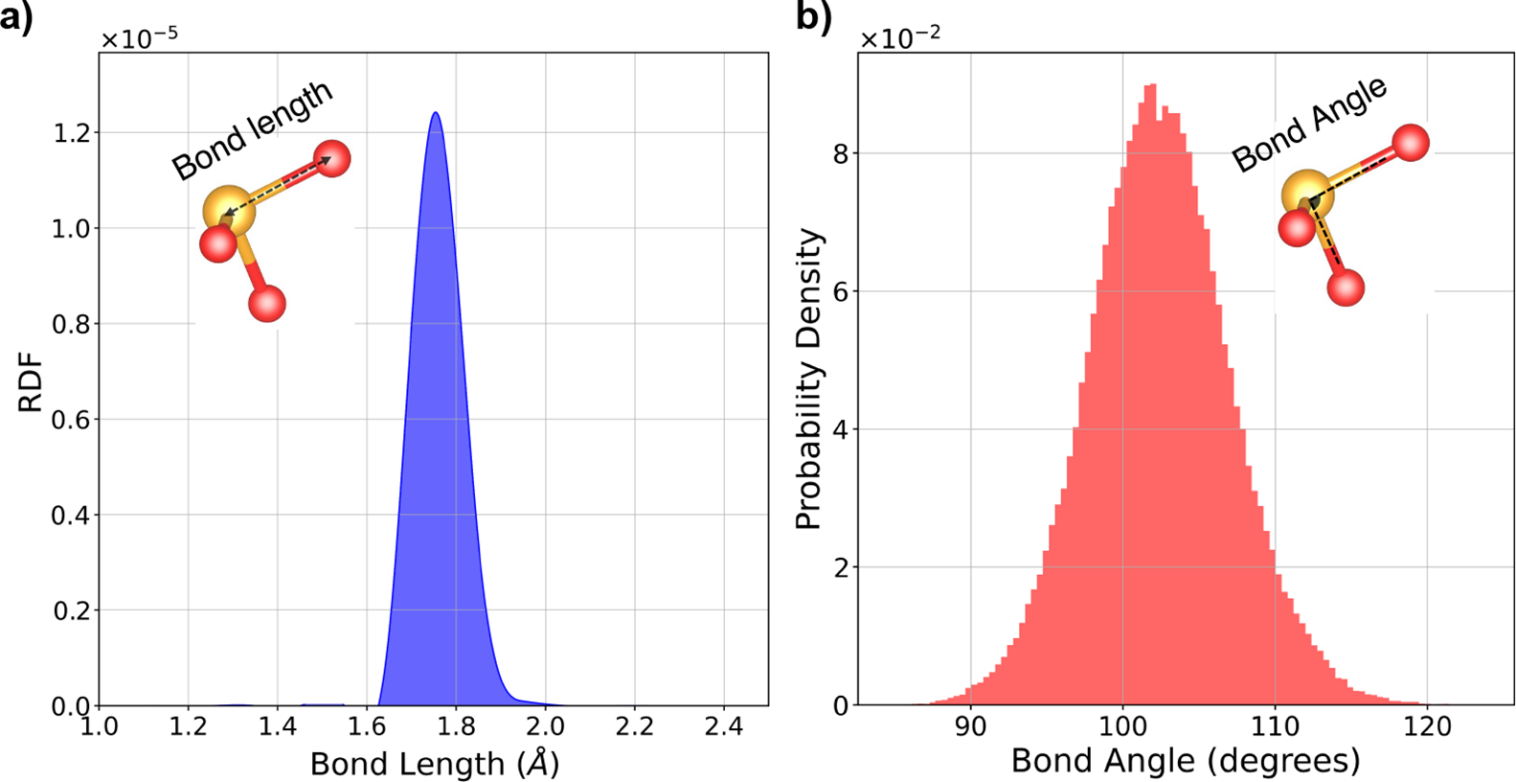
(a) RDF of the Se–O bond length, showing a sharp peak centered around 1.7 Å, indicating a preferred and stable bond length at this distance. (b) Probability density of the Se–O bond angles, displaying a distribution from approximately 90–120°, with a central peak around 102°.

The radial distribution function (RDF) of the Se–O bond length during the MD calculation is extracted to analyze this displacement further. This analysis provides insights into the local structure around a reference particle in an MD simulation. As shown in Figure 7a, there is a sharp peak centered around 1.7 Å, suggesting a preferred bond length at this distance. The RDF indicates a well-defined and stable Se–O bond length. However, examining the probability density of the Se–O bond angles (Figure 7b) reveals that the distribution of bond angles spans from approximately 90–120°, suggesting some variability in bond angles, which might be typical of thermal motion and dynamic interactions. The central peak is around 102°, indicating this compound’s most probable bond angle. This analysis confirms that the negative frequencies of the acoustic phonon branches in the doubled unit cell arise from the variability in the Se–O angles.

The insights gained from the MD simulations about the local behavior of the SeO_3_ tetrahedra in β-Bi_2_SeO_5_ suggest potential localized instabilities at elevated temperatures. Both the thermodynamic and the dynamic stability are well established for Bi_2_SeO_2_ and α-Bi_2_SeO_5_.^31^ The thermal oxidation process at high temperatures results in a dense and stable network of oxygens in α-Bi_2_SeO_5_. However, for β-Bi_2_SeO_5_, thermodynamic instability at elevated temperatures (e.g., transistor operating temperature) might be an issue. Our model for β-oxide is based on previous experimental reports^31,32^ considering the chemical formula of Bi_2_SeO_5_ with a valence state of +4 for Se. However, our detailed MD and DFT studies reveal significant movements of the SeO_3_ tetrahedra, which increase with the temperature and are already substantial at around 450 K (Figure 6b).

While thermal motion generally increases with temperature, the displacements of oxygen atoms in SeO_3_ are notably larger compared to those in the Bi_2_O_2_ framework, as depicted in Figure 6b. This suggests that these movements extend beyond typical thermal displacements, indicating a potential local instability specific to the SeO_3_ units rather than a full phase transition. Additionally, the spectroscopic database^37^ suggests that other Se–O-based compounds, such as SeO_4_ with a valence state of +6 for Se, might exhibit more dynamic and thermodynamic stability compared to SeO_3_ tetrahedra. This opens up the possibility that the composition of interlayer compounds could differ from current experimental observations and warrants further experimental investigation. Supporting this hypothesis, a study on Bi_2_TeO_2_^38^ demonstrates that despite the structural similarity between Bi_2_TeO_2_ and Bi_2_SeO_2_, the oxidized products formed through intercalative oxidation differ. The oxidation of Bi_2_TeO_2_ results in the formation of Bi_2_TeO_6_ (valence state of +6 for Te in the interlayer TeO_4_), while the reported chemical formula resulting from the intercalative oxidation of Bi_2_SeO_2_ is β-Bi_2_SeO_5_. Given that Se and Te are both chalcogens, one might expect similar oxidation behaviors. However, due to selenium’s higher electronegativity and smaller atomic radius, it is likely to be more easily oxidized, potentially reaching higher or at least similar valence states compared to tellurium.

The ease of Se oxidation allows Bi_2_SeO_2_ to oxidize through an intercalative process, preserving the  framework and facilitating the formation of β-Bi_2_SeO_5/6_ at relatively low temperatures without significant atomic rearrangement. In this context, careful control of fabrication conditions (e.g., temperature and oxygen concentration) may stabilize the structure by promoting the formation of stable SeO_4_ interlayer compounds. This suggests that a higher oxidation state of Se, leading to β-Bi_2_SeO_6_, might improve the stability of the oxide.

However, our DFT calculations show that this uncertainty does not significantly affect the intrinsic properties of β-oxide at room temperature. As mentioned earlier, the energy difference between the doubled crystal structure M3 (with more rotated SeO_3_ compounds) compared to M2 is −18.73 meV per atom, given that the thermal energy per atom () at room temperature is about 38.8 meV, the observed energy reduction is relatively small. This implies that a thermal mixture of different SeO_3_ orientations due to thermal fluctuations might be expected at room temperature. This consideration is discussed in detail in the following sections.

### Electronic and Dielectric Properties

**Figure 8:**
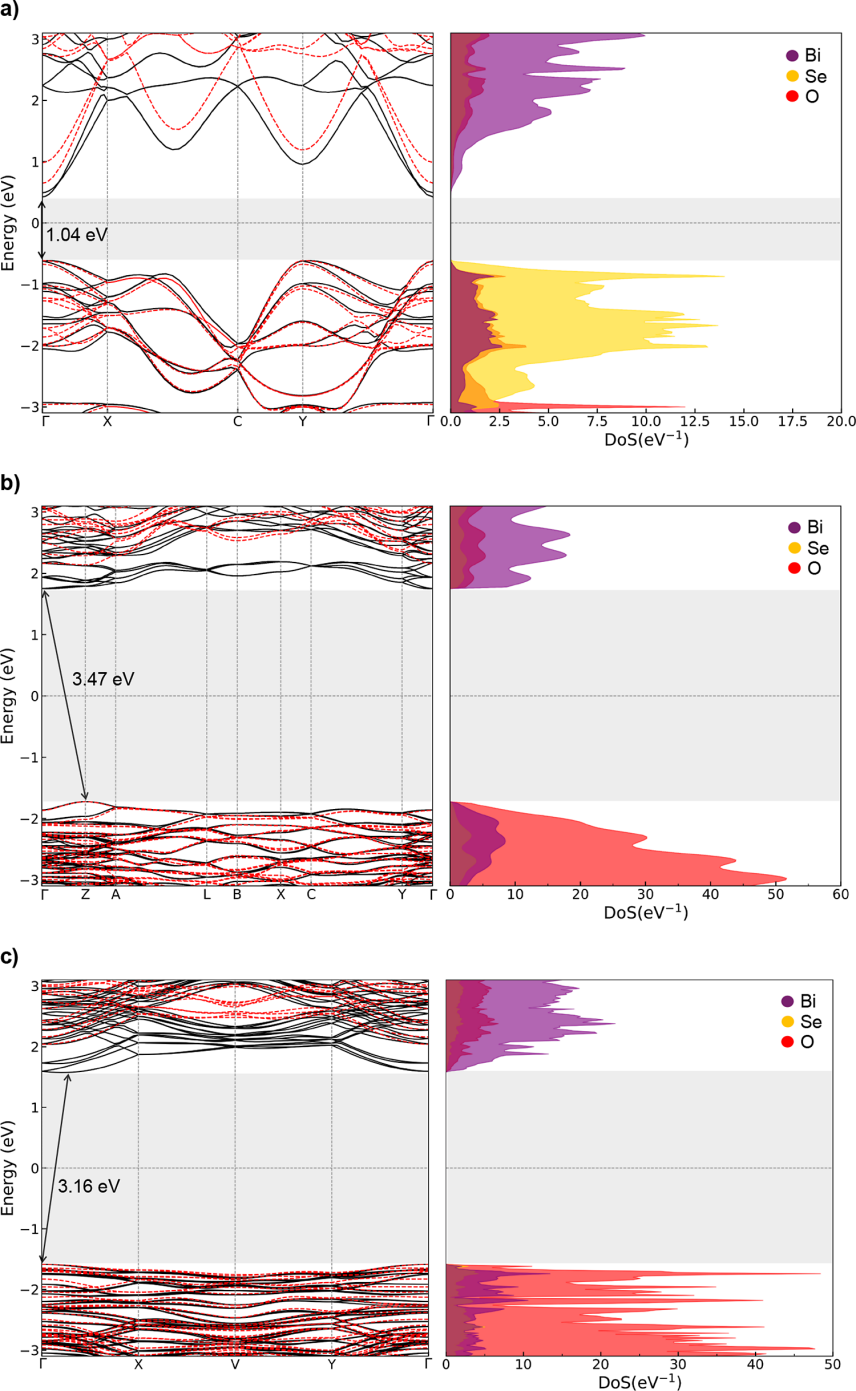
Electronic bandstructure and PDoS of (a) Bi_2_SeO_2_, (b) α-Bi_2_SeO_5_, and (c) β-Bi_2_SeO_5_. The inclusion of SOC (black solid lines) shifts states energetically, reducing the electronic band gap by 220 meV to 1.04 eV for the semiconductor, 420–3.47 eV for α-Bi_2_SeO_5_, and 540–3.16 eV for β-Bi_2_SeO_5_. The valence bands of both oxides are primarily composed of Bi and O atoms, with the O atoms in Bi–O frameworks causing the primary contribution.

The electronic band structure and projected density of states (PDoS) of the Bi_2_SeO_2_ semiconductor and its two native oxides are calculated. Figure 8 demonstrates that spin–orbit coupling (black solid lines) substantially influences the electronic structure of these materials. This quantum mechanical phenomenon arises from the interaction between a particle’s spin and its orbital momentum. For heavy elements, such as Bi, the inner electrons move at speeds that are a significant fraction of the speed of light. This necessitates the inclusion of relativistic effects like spin–orbit coupling (SOC) which are crucial for accurate predictions of the electronic properties.

Figure 8 shows that the semiconductor’s conduction band is primarily composed of orbitals associated with Bi atoms. After including SOC, these states undergo an energetic shift, reducing the electronic band gap of Bi_2_SeO_2_ by 220 meV to 1.04 eV. This effect is more pronounced in the native oxides and results in a band gap reduction from 3.89 to 3.47 eV for α-Bi_2_SeO_5_ and from 3.7 to 3.16 eV for β-Bi_2_SeO_5_. Figure 8b,c indicate that the valence bands of both oxides are composed of Bi and O atoms. However, there are two types of O atoms: those in Bi_2_O_2_ frameworks and those in the SeO_3_ interlayer tetrahedra. Further analysis of the PDoS confirms that the valence bands are mainly composed of O atoms in Bi_2_O_2_ frameworks, indicating that uncertainties in the positions of O atoms in SeO_3_ compounds do not affect the electronic properties of β-Bi_2_SeO_5_.

The effective carrier masses along different directions are extracted by fitting a parabolic dispersion relation near the band extrema. As shown in Table 1, the small isotropic in-plane electron effective masses of Bi_2_SeO_2_ confirm its potential as a high-mobility semiconductor for novel transistors.^39^ On the other hand, both native oxides have anisotropic effective electron masses. In the case of β-Bi_2_SeO_5_, despite a similar crystal structure to that of the semiconductor, slight deviations in the atomic arrangement caused the conduction band minimum to be located between Γ–X (not a high-symmetry point), leading to anisotropic electron effective masses. In addition, the bonding in oxides often involves a more significant ionic character due to the presence of oxygen. The difference in electronegativity between oxygen and the other elements can create anisotropic potential fields, affecting the curvature of the electronic bands. Moreover, both native oxides have larger in-plane effective masses than the semiconductor. While a larger effective mass is generally not desirable for the semiconductor channel of a transistor due to its impact on mobility, it is highly beneficial for the insulating layers since it reduces the tunneling probability. Using a semiconductor with a small effective mass for the channel and an insulator with a larger effective mass is an optimal combination, another benefit of the Bi_2_SeO_2_/Bi_2_SeO_5_ material system.

**Table 1:** Key Material Properties of Bi_2_SeO_2_ and Its Native Oxides

parameter		Bi_2_SeO_2_	α-Bi_2_SeO_5_	β-Bi_2_SeO_5_
effective mass (*m*_0_)		0.15	0.87	1.26
		0.15	0.63	0.96
		0.65	3.08	1.64
		0.20	1.52	1.99
		0.93	2.72	1.99
		17.26	1.1	8.62
dielectric constant (ε_0_)	ε^*a*^	26.65	11.41	10.36
	ε^*b*^	234.02	**35.27**^a^	74.46
	ε^*c*^	**99.48**^a^	14.83	**35.30**^a^
band gap (eV)	*E*_g_	1.04	3.47	3.16

a Out-of-plane permittivity is in bold.

Our calculations show that Bi_2_SeO_2_ and its native oxides exhibit large dielectric constants in both the in-plane and out-of-plane directions, consistent with previously reported values.^31,32,40^ The high out-of-plane dielectric constants in the gate oxides ensure excellent insulating properties and minimize leakage currents for a given EOT. This allows for a thicker gate dielectric layer, improving gate control over the channel and enhancing the overall performance (e.g., subthreshold swing and power consumption) in novel nanoelectronic devices. Additionally, the high dielectric constant of the Bi_2_SeO_2_ semiconductor enhances the charge carrier mobility through the screening of scattering mechanisms, such as those caused by ionized impurities (Coulomb centers) or polar phonons from adjacent dielectrics or substrates.^9^ However, high in-plane dielectric constants also present challenges. A high in-plane dielectric constant in the semiconductor can lead to stronger electrostatic coupling between the drain and the channel, potentially increasing the short-channel effects. Similarly, a high in-plane dielectric constant in the oxide can result in stronger parasitic electrostatic control from the drain to the channel, potentially reducing the effectiveness of gate control. These factors necessitate careful transistor design considerations to mitigate these effects such as dual-gate, FinFET, or GAA structures.

**Figure 9:**
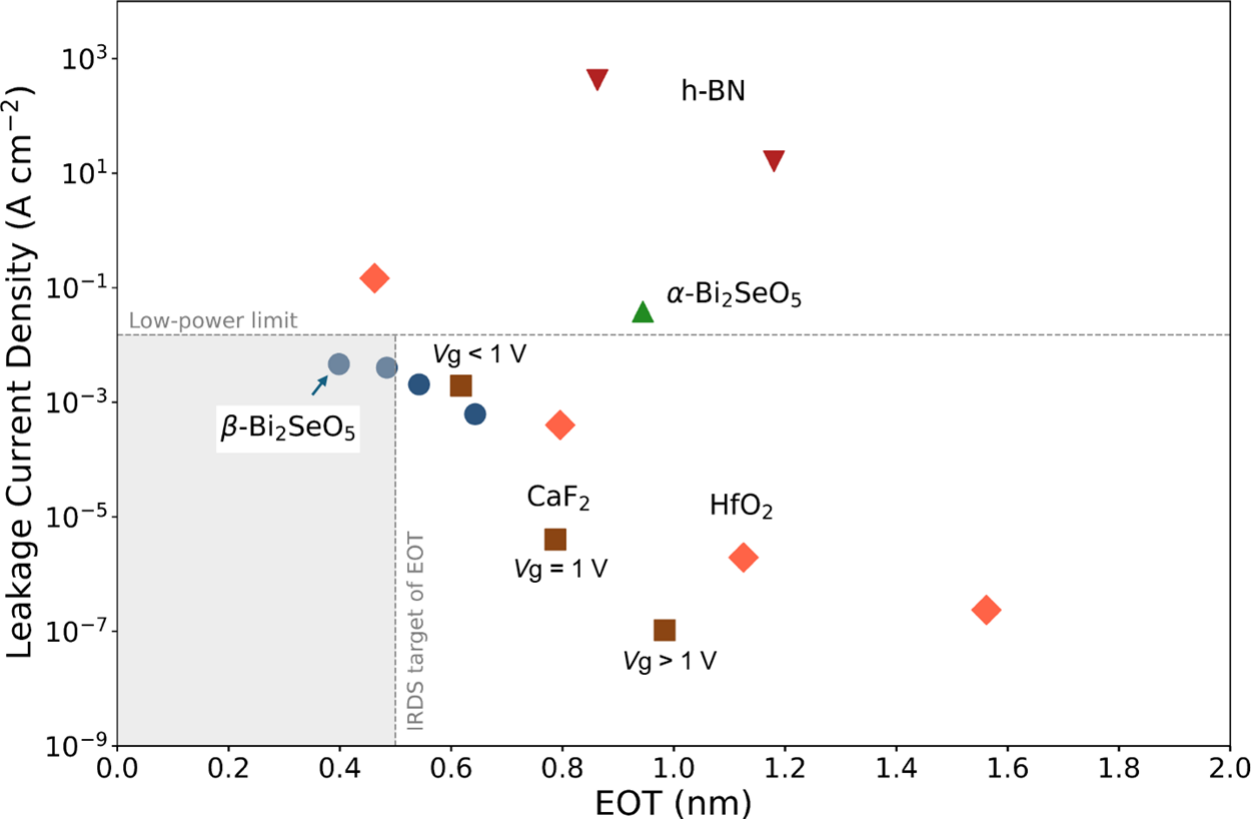
Comparison of reported measured leakage current density versus EOT for various dielectric materials, including α-Bi_2_SeO_5_,^30^ β-Bi_2_SeO_5_,^31^ 2D hBN,^41^ single-crystalline CaF_2_,^42^ and ALD-synthesized HfO_2_.^23,43^ Leakage currents are measured at a gate voltage of 1 V, except for CaF_2_. The β-Bi_2_SeO_5_ achieves leakage current densities below the low-power limit of 0.015 A/cm^2^ while meeting the IRDS target EOT of sub-0.5 nm (gray area).

Finally, the measured dielectric properties of various insulators, including α-Bi_2_SeO_5_,^30^ β-Bi_2_SeO_5_,^31^ 2D hBN,^41^ single-crystalline CaF_2_,^42^ and ALD-synthesized HfO_2_,^23,43^ are reported in Figure 9. Furthermore, LaOCl has recently been identified as a promising insulator^44,45^ with a low leakage current at an EOT of 18.9 nm (determined for its lowest reported experimental thickness of 23.8 nm, according to Figure S9 and Table S1 of the Supporting Information^44^). Among these, single-crystalline β-Bi_2_SeO_5_ demonstrates superior performance with leakage currents below the low-power limit of 0.015 Acm^–2^ and an EOT of less than 0.5 nm, meeting IRDS 2021 standards.^4^ This performance arises from its intrinsic properties and high structural integrity with the underlying high-mobility 2D semiconductors, making it suitable for advanced electronics with improved scalability and power efficiency. While the α-phase shares similar intrinsic properties with the β-phase and is thermodynamically stable at high temperatures, its higher achievable EOT (0.9 nm) and increased leakage currents restrict its suitability for scaled devices. These differences are largely due to the distinct fabrication processes, resulting in polycrystalline vs crystalline structures for the α- and β-phases, respectively. Moreover, according to previous experimental studies, the dielectric properties of α-Bi_2_SeO_5_ remain stable down to 5 nm thickness but change below this limit.^30^ The polycrystalline nature of the α-phase, alongside a lower interface quality with 2D semiconductors due to lattice mismatching, introduces grain boundaries, defects, and traps, ultimately degrading the device performance. Consequently, β-Bi_2_SeO_5_ is better suited for high-performance scaled devices, while α-Bi_2_SeO_5_ may be preferable for high-temperature applications.

### Semiconductor/Oxide Heterostructure

One of the key advantages of high-mobility Bi_2_SeO_2_ compared to other well-studied 2D semiconductors like MoS_2_ is the existence of compatible crystalline native oxides. As discussed, these native oxides are formed through layer-by-layer oxidation of the underlying semiconductor in a nondestructive manner.

**Figure 10:**
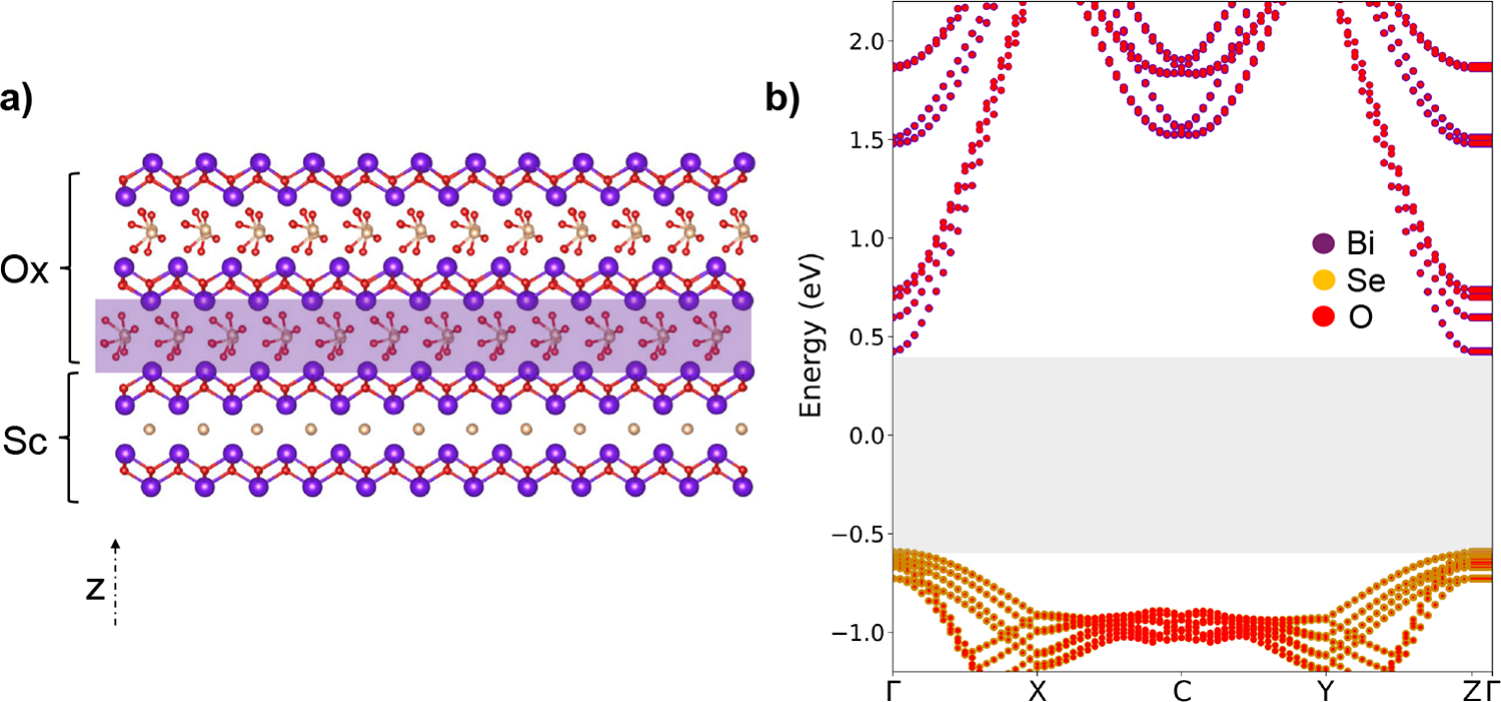
(a) Semiconductor-oxide heterostructure. The shaded rectangle is the interface between the semiconductor and the oxide. (b) Projected electronic bandstructure of the heterostructure. The gray region indicates a clean bandgap without surface states.

To comprehensively understand the intrinsic properties of these semiconductor-oxide combinations, modeling a heterostructure based on these materials is essential. However, this requires suitable surface terminations for both the semiconductor (Bi_2_SeO_2_) and oxide (Bi_2_SeO_5_) in the model. Due to the unique zipper-like nature of these materials (Figure 2), it is important to prevent nonphysical effects in modeling their surfaces. The surface terminations of each material, either Bi–O or Se, are analyzed as they significantly influence the surface chemistry and electronic properties of the semiconductor and the oxide. Our DFT simulations indicate that a Se termination retains the basic electronic properties of the material more effectively and thus was used for subsequent studies. Slabs representing finite-sized segments of the respective crystals included multiple layers to capture the characteristics accurately. Bulk Bi_2_SeO_2_ crystals, when cleaved along the *c*-axis, reveal a non-neutral layered structure, similar to an opened fractured zipper, leaving some Se atoms bonded to each Bi–O layer (see Figure 2b). High-resolution STM studies confirm the tendency of Se atoms and surface vacancies to dimerize, forming distinctive 2× *n* patterns consistent with previous works.^33^ Additionally, the interface between the semiconductor and the oxide must be determined to create the heterostructure. This consists of aligning lattice parameters and orienting crystal structures to prepare a coherent interface. Our DFT calculations suggest that the simplest interface between semiconductor and oxide is constructed by the oxide surface (shaded rectangle in Figure 10a), resulting in an atomically sharp interface. The projected electronic band structure of the resulting heterostructure is shown in Figure 10b. As can be seen in the gray region, the heterostructure possesses a clean bandgap, created at the interface by, for example, introducing surface dipoles. This structural integrity is crucial for preserving the desirable electronic properties of the material, making it suitable for reliable nanoelectronic applications.

**Figure 11:**
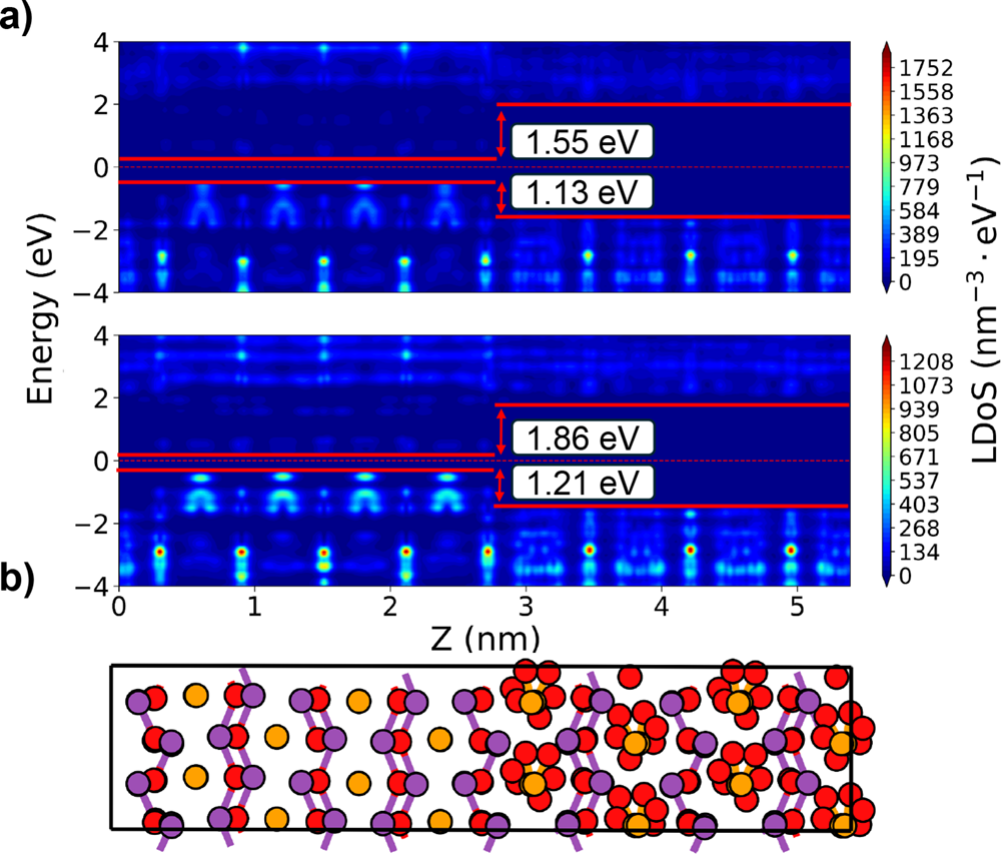
LDoS along the *z*-axis for two heterostructures with (a) compressive strain on the oxide crystal and (b) tensile strain on the semiconductor. Both heterostructures exhibit Type I band alignment.

The different lattice parameters of Bi_2_SeO_2_ and its oxides, for example, β-Bi_2_SeO_5_, necessitate consideration of potential strain at the interface, as the semiconductor and oxide are not completely lattice-matched. The local Density of States (LDoS) along the *z*-axis is calculated for two heterostructures (Figure 11). The key difference is whether the strain is applied to the semiconductor or the oxide to enforce a commensurate lattice for both materials. Figure 11a shows the LDoS with compressive strain on the oxide crystal, while Figure 11b represents the heterostructure with tensile strain on the semiconductor. The band alignment between the semiconductor and oxide shows a conduction band offset of 1.55 and 1.86 eV, and a valence band offset of 1.13 and 1.21 eV for the heterostructures with strain on the oxide and semiconductor, respectively. Both heterostructures exhibit a Type I band alignment, advantageous for the semiconductor-oxide material stack, facilitating efficient confinement of electrons and holes within the semiconductor, and leading to low leakage current in transistors based on this heterostructure.

## Conclusions

We comprehensively analyzed the intrinsic properties of the 2D semiconductor Bi_2_SeO_2_, its native oxides, and its interfaces, underlining their role as promising candidates for future nanoelectronics. Our DFT and MD investigations confirmed their promising structural, electronic, and dielectric properties. We demonstrated that the smooth, non-vdW interface between Bi_2_SeO_2_ and its native oxide, β-Bi_2_SeO_5_, combined with a high dielectric constant (>30) and favorable band alignment (1.13 eV for holes and 1.55 eV for electrons) and the reasonably wide bandgaps of its native oxides, with 3.47 eV for α-Bi_2_SeO_5_ and 3.16 eV for β-Bi_2_SeO_5_, position this semiconductor–insulator pair as a formidable contender for future transistor technologies. We thoroughly discussed the determination of the crystal structure of β-Bi_2_SeO_5_ and analyzed its stability. The intrinsic properties identified in this study represent the highest achievable properties for these materials; however, real materials will be impacted by defects, which will be the subject of future studies. Our insights provide a theoretical basis for future research and development, facilitating further enhancements and practical implementations of Bi_2_SeO_2_-based transistors.

## Methods

### Computational Methods

In our comprehensive first-principles study, we employed various structures to investigate different properties. To leverage the strengths of each software package, we used a combination of computational tools, ensuring precise and efficient simulations across the different scales of our study. DFT simulations are conducted using Quantum-ATK for structural (e.g., lattice parameters, crystal symmetry, and bonding) and electronic properties.^46^ We utilized a localized basis set within the linear combination of atomic orbitals (LCAO) approach with PSEUDODOJO pseudopotentials.^47^ Calculations used a k-point sampling density of 8 Å and a density mesh cutoff of 125 Ha. SOC is included to account for the relativistic effects. To overcome typical shortcomings of semilocal DFT like the common underestimation of electronic bandgaps, we employed the hybrid functional HSE06. Dispersion corrections are applied using the Grimme DFT-D3 method to account for vdW interactions.^48^ Structural optimizations continued until the interatomic forces were less than 0.01 eV/Å and the unit cell pressure was below 0.1 GPa.

For simulations requiring large supercells, such as phonon calculations or MD simulations, we utilized the highly efficient CP2K^49^ software package due to its superior performance in handling large systems. MD simulations are performed at temperatures up to 450 K. The electronic structure is described using DZVP-MOLOPT-SR-GTH basis sets and GTH-PBE pseudopotentials,^50^ with density fitting facilitated by the FIT9 auxiliary basis set. The Auxiliary Density Matrix Method (ADMM) was employed within the CP2K framework to enhance the computational efficiency. Simulations ran for 4480 steps with a time step of 1 fs. Phonon calculations involved tighter structural optimization. Geometry and cell optimizations were performed using the LBFGS optimizer, employing a maximum force criterion of 1 × 10^–5^ Ha/Bohr and a pressure tolerance of 0.01 GPa. A high energy cutoff of 400 Ha and a convergence criterion of 5 × 10^–10^ Ha were applied for accurate force calculations. The fully optimized structure was then processed using the Phonopy package,^35^ where supercells of dimensions 4 × 4 × 2 were generated with atomic displacements of 0.02 Å for the vibrational analysis. These small, finite displacements from equilibrium positions were used to calculate the resulting forces via DFT. The displacement forces were computed with CP2K, enabling an accurate determination of the force constants. These constants were then used to construct the dynamic matrix from which phonon frequencies and eigenvectors were derived to assess the stability of the structure. The acoustic sum rule correction was applied to ensure momentum conservation throughout the calculations.

The dielectric tensor was calculated using the finite electric field method combined with the Berry phase approach,^51,52^ capturing both the electronic and ionic contributions. The electronic contribution was determined by evaluating the polarization response of the electronic structure under an external electric field. To account for the ionic contribution, the Born effective charges were calculated to measure the coupling between atomic displacements and changes in polarization. This combined approach accurately represents the vibrational properties of the material, particularly for polar systems in which long-range Coulomb interactions play a significant role in influencing the dielectric behavior. These long-range Coulomb interactions manifest in the phonon dispersion as LO–TO splitting near the zone center (*q* = 0). In polar materials, distinguishing between longitudinal optical (LO) and transverse optical (TO) phonon modes is crucial for capturing anisotropic responses. This LO–TO splitting occurs due to the coupling between the ionic lattice and macroscopic electric fields, leading to different phonon frequencies for the LO and TO modes. While TO modes primarily contribute to the low-frequency dielectric response through ionic displacements in response to slowly varying electric fields, LO modes are more associated with the material’s response at optical frequencies (high-frequency dielectric constant due to the electron response to rapidly oscillating electric fields). Properly accounting for LO–TO splitting is essential for high-κ materials, as it directly impacts the material’s anisotropic response to an external perturbation. To account for LO–TO splitting in the phonon calculations, a nonanalytical term correction^53,54^ was introduced into the dynamical matrix. This correction ensures that long-range Coulomb interactions are accurately represented, particularly for LO phonons near the zone center.

### Cross-Sectional STEM Characterization

A focused ion beam (FIB) sample was fabricated using an FEI Scios 2 Dual Beam SEM/FIB system that adhered to standard FIB preparation protocols. To investigate the detailed structure of layered Bi_2_SeO_5_, cross-sectional samples were prepared along the (100) and (110) lattice planes. The atomic structure of Bi_2_SeO_5_ was examined using cross-sectional STEM. Atomic-resolution bright field (BF) and integrated differential phase contrast (iDPC) images were obtained using an aberration-corrected scanning transmission electron microscope (AC-STEM) in an FEI Titan Cubed Themis G2 300, operated at an acceleration voltage of 300 kV, to determine the position of both oxygen and bismuth/selenium atoms.

## Notes

This manuscript was previously submitted to the ChemRxiv preprint server. The citation for the preprint is as follows: P.K.; D.W.; M.B.; T.K.; M.P.; M.R.D.; Y.Z.; X.G.; H.P.; M.W.; T.G. Two-dimensional Bi_2_SeO_2_ and Its Native Insulators for Next-Generation Nanoelectronics. 2024, chemrxiv-2024-72gpv. ChemRxiv. 10.26434/chemrxiv-2024-72gpv (accessed November 8, 2024).
